# SeLECTS-assisted identification based on structured EEG reports: Development and external validation of a multicenter machine learning model

**DOI:** 10.1097/MD.0000000000049480

**Published:** 2026-07-03

**Authors:** Lijun Li, Lingxiang Ao, Lei Li, Li Wang, Xiaomei Liu, Kai Liu

**Affiliations:** a Neurophysiology Center, Kunming Children’s Hospital & Children’s Hospital of Kunming Medical University, Kunming, China; b Department of Quality and Safety, Kunming Children’s Hospital & Children’s Hospital of Kunming Medical University, Kunming, China; c Comprehensive Pediatrics, Kunming Children’s Hospital & Children’s Hospital of Kunming Medical University, Kunming, China.

**Keywords:** BECT, calibration curve, decision curve, external validation, LASSO, SHAP, XGBoost

## Abstract

Benign epilepsy with centrotemporal spikes (SeLECTS) is a common pediatric epilepsy characterized by high-amplitude central–temporal spikes on electroencephalogram (EEG). Early identification remains challenging owing to inter-reader variability and inter-center protocol differences. Existing models often rely on single-center raw EEG, limiting generalizability and clinical deployment. An explainable, cross-institutional tool built on structured EEG reports plus routine laboratory tests could improve screening and workflow. This study aimed to develop a machine learning model for SeLECTS assistance and validate its performance on an external dataset, comparing multiple algorithms and evaluating discrimination, calibration, and net clinical benefit, with interpretable outputs for quality control and patient stratification. We performed a multicenter retrospective study (internal n = 1067; external n = 94). Among 242 candidate variables, those with >20% missingness were removed; remaining missing data were handled by multiple imputation. Least Absolute Shrinkage and Selection Operator selected 13 key features, including age; central/temporal discharge localization; spikes or spike–wave complexes; background rhythm; facial/limb clonus; and laboratory indices (e.g., lactate dehydrogenase, white blood cell differential, 25-hydroxyvitamin D). Ten common classifiers were trained and tested. Area Under the Receiver Operating Characteristic Curve (AUROC) was the primary metric; accuracy, sensitivity, specificity, and F1 score were secondary. Calibration and decision curve analyses assessed probability agreement and clinical net benefit. SHapley Additive exPlanations provided global and patient-level interpretability. eXtreme Gradient Boosting performed best. Internal validation yielded AUROC 0.97, accuracy 0.91, sensitivity 0.83, and specificity 0.94. External validation showed AUROC 0.96, accuracy 0.88, sensitivity 0.74, and specificity 0.94. Compared with internal performance, external results declined only slightly (AUROC −0.01; accuracy −0.03; sensitivity −0.09; specificity unchanged). Calibration indicated close agreement between predicted and observed risks. Decision curve analysis showed consistent positive net benefit across low-to-moderate thresholds. SHapley Additive exPlanations highlighted temporal discharges, age, central discharges, and Lactate Dehydrogenase as leading contributors, consistent with known SeLECTS characteristics. Using 13 multicenter, routinely available features, the eXtreme Gradient Boosting model demonstrated high discrimination, good calibration, and meaningful net benefit in internal and external cohorts. Designed as a second-view aid after EEG reporting, the interpretable, lightweight model supports risk alerts and tiered review and is suitable for scalable deployment across centers.

## Graphical abstract

**Figure:**
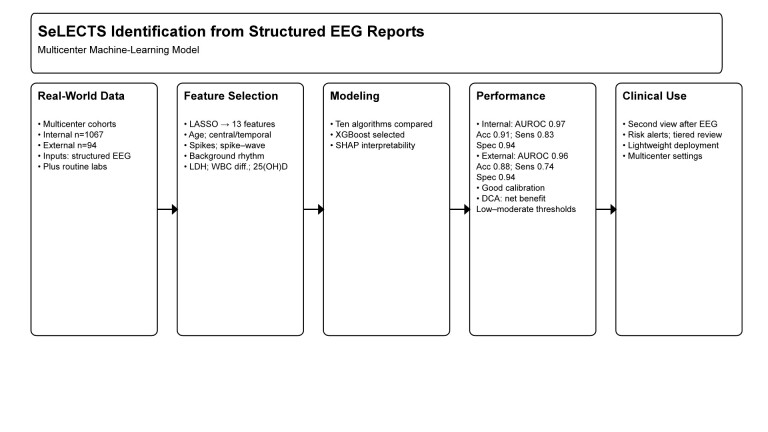

## 1. Introduction

Self-limited epilepsy with centrotemporal spikes (SeLECTS; traditionally termed Benign Epilepsy with Centrotemporal Spikes (BECT)/Rolandic epilepsy) is one of the most common self-limiting epilepsy syndromes in childhood.^[1]^ It is characterized by sleep-related oropharyngeal and facial sensory-motor seizures with speech impairment, accompanied by high-amplitude centrotemporal spikes/spike-and-wave discharges on electroencephalogram (EEG).^[2,3]^

Despite a generally favorable prognosis, SeLECTS exhibits marked heterogeneity in age of onset, seizure lateralization, seizure burden, and associated attention or learning difficulties.^[4–7]^ Cognitive, behavioral, and linguistic disturbances have been increasingly recognized in both active and remission phases.^[8–11]^ Some children present atypically at initial diagnosis, leading to delayed classification that impacts clinical follow-up and family decision-making.^[12]^

Current clinical identification relies primarily on history, physical examination, and EEG. However, EEG interpretation is influenced by reader experience, recording parameters, and inter-center workflow variability, while limited neurophysiology staffing in primary pediatric care exacerbates delays and repeat testing.^[13,14]^ Therefore, clinicians require a reproducible, transferable, and workflow-friendly auxiliary identification tool that extracts SeLECTS-related key patterns from multi-source structured information to enhance initial triage and follow-up stratification efficiency.^[15,16]^

In recent years, machine learning (ML) approaches have been increasingly applied to EEG-based seizure detection/prediction and epilepsy screening, and prior studies generally report strong discrimination and/or high accuracy across different feature settings.^[17–19]^ Among commonly compared algorithms, tree-ensemble methods: particularly gradient-boosted trees such as eXtreme Gradient Boosting (XGBoost): are attractive because they capture nonlinear interactions and perform well with mixed structured predictors, making them practical candidates for workflow-integrated decision support.^[18,19]^ Nevertheless, ML studies focusing on SeLECTS remain largely constrained by single-center cohorts, limited sample sizes, heterogeneous feature definitions, and insufficient evaluation of external validity, calibration, clinical net benefit, and interpretability, which collectively weakens the real-world evidence base for deploying such models (including XGBoost) in routine practice.^[20,21]^

Addressing these clinical challenges and methodological gaps, this study proposes to: select key features using regularization and embedding methods based on structured variables from multicenter electronic medical records and EEG reports; compare multiple algorithms and systematically evaluate models’ comprehensive performance in discriminative ability (Area Under the Receiver Operating Characteristic Curve [AUROC]/Area Under the Precision–Recall [PR] Curve), calibration, and clinical net benefit; introduce external data to validate cross-institutional generalization; and explore interpretability and feasibility for online application. Research objectives are: to construct the SeLECTS-assisted identification model for clinical settings and identify its key features; to validate model robustness, calibration, and clinical gain through internal and external validation; and to provide a lightweight tool embeddable into outpatient, emergency, and follow-up settings without altering existing examination pathways.

## 2. Methods

### 2.1. Research design and ethics

This study is a multicenter, retrospective diagnostic predictive study aimed at constructing ML models for clinical decision support in BECT/SeLECTS. As the study is retrospective, informed consent is waived; ethical review and data de-identification follow each center’s standard procedures.

### 2.2. Data sources and study population

#### 2.2.1. Internal training/validation data

Derived from the EEG reporting system and electronic medical records of the Neurophysiology Center at Kunming Children’s Hospital, covering the period from 2018 to 2024.

#### 2.2.2. External validation data

Comparable data from multiple prefectural hospitals (e.g., Honghe Prefecture People’s Hospital, Wenshan Prefecture People’s Hospital, Yanshan County People’s Hospital, Hekou County People’s Hospital, Puer City Women and Children’s Hospital) collected from 2018 to present, used to evaluate the model’s cross-institutional generalization performance.

#### 2.2.3. Inclusion criteria

Pediatric patients (≤16 years) who underwent routine EEG evaluation and had a finalized structured EEG report with sufficient accompanying clinical record for outcome adjudication.

#### 2.2.4. Exclusion criteria

Age > 16 years; incomplete baseline EEG-report information that precludes outcome determination.

#### 2.2.5. Outcome definition and reference standard

The prediction target was a binary outcome (SeLECTS/BECT vs non-SeLECTS). The reference standard was the finalized diagnosis documented in the structured EEG report and corresponding medical record, made by qualified neurophysiology physicians following guideline-consistent electroclinical criteria. The non-SeLECTS cases within the same source population served as the comparator (negative) class. A direct“human vs ML”head-to-head comparison would require an independent blinded multi-reader reinterpretation (or standardized manual scoring) of raw EEG separate from the original report; moreover, using prior clinical decisions from the same record as a comparator would not be methodologically independent and may introduce incorporation bias.

#### 2.2.6. External validation diagnostic criteria

EEG diagnoses based on the latest EEG clinical guidelines (consistent with internal criteria).

#### 2.2.7. Data volume corresponding to results

Internal *n* = 1067; External Validation *n* = 94 (for independent model universality testing).

### 2.3. Variables and data collection

As described in the Results section, the following information was systematically extracted for all included cases:

#### 2.3.1. Demographic and clinical history

Age, gender, chief complaint, medical history, etc;

#### 2.3.2. EEG report hierarchy variables

Central/temporal localization, spikes, spike-and-wave complexes, background rhythms, etc (including lateralization, presence/absence of recording, and site);

#### 2.3.3. Laboratory and routine tests

Complete blood count with differential, arterial blood gas analysis, liver/kidney function, electrolytes, coagulation parameters, etc (extracted based on actual availability).

### 2.4. Data processing and missing value handling

To reduce bias caused by missing values and maintain consistency with results:

Exclude candidate factors with missing values exceeding 20%;

Apply multiple imputation (Multivariate Imputation by Chained Equations) for missing values in the remaining variables; Imputation is performed on the modeling data before proceeding to subsequent feature selection and fitting.

### 2.5. Feature selection

Feature selection was performed to reduce dimensionality and mitigate overfitting. After excluding candidate variables with >20% missingness and imputing the remaining missing values using Multivariate Imputation by Chained Equations, we applied L1-regularized (Least Absolute Shrinkage and Selection Operator [LASSO]) logistic regression to the full candidate set (242 variables), including structured EEG-report descriptors, clinical semiology variables, and routinely available laboratory indices.

The penalty parameter (λ) was selected using 10-fold cross-validation on the internal training data by minimizing the mean cross-validated loss (λ-min). The resulting sparse solution yielded 13 features (age; central/temporal regions; facial/limb twitching; background; spike-and-wave complex; spikes; Lactate Dehydrogenase (LDH); eosinophil percentage; neutrophil percentage; basophil count; and 25-hydroxyvitamin D), which were fixed and carried forward unchanged for subsequent model development and interpretation.

### 2.6. Model construction and evaluation process

#### 2.6.1. Data splitting and validation strategy

The internal cohort was split using stratified random sampling into training/validation/test subsets to preserve outcome proportions. The validation subset was used for model selection, hyperparameter tuning, and decision-threshold specification, whereas the held-out test subset was used for final internal performance estimation. The external cohort served as an independent external validation set and was not used for feature selection, tuning, or refitting.

#### 2.6.2. Candidate algorithms and rationale for selecting XGBoost

We compared ten classifiers consistent with the Results (Logistic Regression, Support Vector Machine, Decision Tree, Random Forest, GBM, k-Nearest Neighbors, Naive Bayes, Multi-Layer Perceptron, AdaBoost, and XGBoost). Given the mixed structured EEG-report variables and laboratory indices (tabular predictors with expected nonlinearities and interactions), we selected the final model based on a joint assessment of discrimination, calibration, and clinical net benefit; XGBoost showed the most favorable overall profile and was retained as the final application prototype.

#### 2.6.3. Handling class imbalance and overfitting risk

To address potential class imbalance, we used stratified splitting and algorithm-level weighting (e.g., class weights for linear models and scale_pos_weight-style weighting for boosted trees when applicable). Overfitting was mitigated by LASSO-based feature reduction, confining tuning to training/validation only, evaluating on a held-out test set, and confirming transportability on external validation.

#### 2.6.4. Performance metrics and reporting

AUROC was the primary metric. We additionally reported Area Under the PR Curve, accuracy, sensitivity, specificity, precision, recall, F1 Score (harmonic mean of precision and recall), PPV/NPV, and confusion matrices for internal test and external validation. Calibration was assessed using calibration curves, and clinical utility was evaluated using decision curve analysis. Statistical modeling was conducted in Python 3.10 with two-sided *P* < .05.

#### 2.6.5. Sample size considerations and descriptive statistics

This multicenter study was retrospective, and the sample size was therefore determined by the number of eligible consecutive cases available during the study period (internal cohort n = 1067; external validation n = 94). Accordingly, a priori power analysis for hypothesis testing was not performed. To minimize overfitting under a fixed sample size, we reduced the feature space to 13 predictors via LASSO, restricted model selection/tuning to the training/validation data only, and estimated performance on a held-out test set with confirmation in the independent external cohort. Table 1 reports numeric descriptive summaries for all variables (mean, SD, minimum/maximum, and sample size); for binary predictors encoded as 0/1, the mean corresponds to the proportion with the feature present.

**Table 1:** Population baseline information form.

Characteristic	Mean	Standard deviation	Minimum	Maximum	Sample size
Gender	0.59	0.492	0	1	1067
Age	2847.123	869.266	317	6041	1067
**Electroencephalogram**					
Right	0.432	0.496	0	1	1067
Frontal pole	0.009	0.096	0	1	1067
Frontal	0.105	0.307	0	1	1067
Central	0.79	0.407	0	1	1067
Parietal	0.862	0.345	0	1	1067
Occipital	0.238	0.426	0	1	1067
Is there an alpha rhythm	0.878	0.327	0	1	1067
Is there a theta rhythm	0.884	0.321	0	1	1067
Low-amplitude fast waves interspersed	0.999	0.031	0	1	1067
Approximately symmetrical on both sides	0.998	0.043	0	1	1067
Modulation amplitude modulation	0.886	0.318	0	1	1067
Bilateral	0.439	0.496	0	1	1067
Left	0.407	0.491	0	1	1067
Temporal	0.957	0.203	0	1	1067
Anterior temporal	0.107	0.309	0	1	1067
Middle temporal	0.052	0.223	0	1	1067
Temporal region	0.823	0.382	0	1	1067
Midline	0.151	0.358	0	1	1067
Lower temporal	0.01	0.101	0	1	1067
Anterior head	0.007	0.081	0	1	1067
Posterior head	0.007	0.086	0	1	1067
Spike	0.966	0.181	0	1	1067
Spike-slow complex	0.964	0.185	0	1	1067
Multiple spikes	0.047	0.211	0	1	1067
Multiple spike-and-slow waves	0.199	0.399	0	1	1067
Spike	0.035	0.183	0	1	1067
Spike-shaped slow wave	0.002	0.043	0	1	1067
Spike-wave rhythm	0.003	0.053	0	1	1067
Fast waves	0.003	0.053	0	1	1067
Is the background amplitude normal	0.051	0.219	0	1	1067
Is the background frequency normal	0.108	0.31	0	1	1067
Background	0.143	0.351	0	1	1067
**Clinical presentation**					
Mouth corner deviates to one side	0	0	0	0	1067
Facial twitching	0.006	0.075	0	1	1067
Snoring	0	0	0	0	1067
Drooling	0.142	0.35	0	1	1067
Slurred speech	0.066	0.248	0	1	1067
Confusion	0.007	0.086	0	1	1067
Convulsions	0.661	0.474	0	1	1067
Ipsilateral upper limb	0	0	0	0	1067
Hand and upper limb convulsions	0.317	0.465	0	1	1067
Convulsions in all limbs	0.701	0.458	0	1	1067
Increased discharges during sleep	0.865	0.342	0	1	1067
**Laboratory test results**					
25-hydroxyvitamin D	4.827	19.437	0	157	1067
C-reactive protein	0.036	1.067	0	34.81	1067
pH	0.155	1.019	0	8	1067
PT (prothrombin time)	0.008	0.088	0	1.08	1067
TSH thyroid-stimulating hormone	0.543	1.393	0	9.62	1067
Alpha-hydroxybutyric acid	99.475	86.254	0	265	1067
Gamma-Glutamyl transferase	8.452	8.887	0	76	1067
Triiodothyronine	0.394	0.883	0	3.1	1067
Alanine aminotransferase	8.754	10.697	0	119	1067
Hepatitis C virus antibody	0	0.015	0	0.5	1067
Neutrophil percentage	27.825	23.7	0	90.8	1067
Neutrophil count	1.957	2.021	0	14.69	1067
Toxic granules	0.001	0.022	0	0.5	1067
Medium Fluorescent Reticulocytes	0.004	0.116	0	3.8	1067
Hepatitis B virus e antibody	0.004	0.046	0	0.5	1067
Hepatitis B virus e antigen	0.004	0.046	0	0.5	1067
HBc antibody	0.004	0.046	0	0.5	1067
Hepatitis B surface antigen	0.004	0.046	0	0.5	1067
Treponema pallidum antibody	0	0.015	0	0.5	1067
Human immunodeficiency Virus antibody types 1 + 2	0	0.015	0	0.5	1067
Lactate dehydrogenase	142.219	123.288	0	388	1067
Lactate Dehydrogenase Isoenzymes	30.106	26.195	0	83	1067
Partial pressure of carbon dioxide	0.109	1.779	0	30.3	1067
Low-fluorescence reticulocytes	0.183	4.226	0	99.5	1067
Partial pressure of carbon dioxide at body temperature	0.081	1.524	0	30.3	1067
Shunt index at body temperature	0.02	0.476	0	13.5	1067
Erythropoietin	0.103	1.704	0	36.2	1067
Adrenocorticotropic hormone	0.904	5.885	0	99.42	1067
Immunoglobulin A (serum)	0.02	0.193	0	2.72	1067
Immunoglobulin G	0.147	1.299	0	16.74	1067
Immunoglobulin M (serum)	0.02	0.182	0	2.56	1067
Total Carbon Dioxide Concentration in Whole Blood	0.1	1.887	0	38	1067
Prothrombin time (stago)	0.088	1.084	0	13.8	1067
Prothrombin time control	0.103	1.187	0	14.8	1067
Thrombin time (stago)	0.118	1.452	0	18.8	1067
Shunt index	0.02	0.476	0	13.5	1067
Arterial-to-alveolar oxygen partial pressure ratio	0.234	4.457	0	98.6	1067
Arterial blood oxygen compensation factor	0.004	0.083	0	1.8	1067
Percentage of monocytes	3.699	3.343	0	16.1	1067
Monocyte count	0.248	0.24	0	1.48	1067
Folic acid	0.121	1.898	0	40	1067
Oxygen concentration	0	0.006	0	0.21	1067
Respiratory index	0.061	1.409	0	38	1067
Percentage of basophils	0.117	0.182	0	3	1067
Basophil count	0.008	0.011	0	0.1	1067
Eosinophil percentage	1.661	2.207	0	20.9	1067
Eosinophil count	0.107	0.147	0	1.58	1067
Large platelet ratio	13.012	11.973	0	50.5	1067
Aspartate aminotransferase	17.794	15.327	0	83	1067
Aspartate Aminotransferase/Alanine Aminotransferase	1.458	1.376	0	11.33	1067
Actual base excess	-0.017	0.334	-9	0	1067
Actual bicarbonate	0.049	0.928	0	18.8	1067
Urea	2.825	2.47	0	11.08	1067
Uric acid	183.2	157.195	0	644	1067
Mean corpuscular volume	51.168	40.619	0	95	1067
Mean corpuscular hemoglobin	17.492	13.902	0	33	1067
Mean corpuscular hemoglobin concentration	210.454	166.546	0	376	1067
Mean platelet volume	5.792	4.699	0	13.3	1067
Atypical lymphocytes	0.001	0.026	0	0.5	1067
Total cholesterol	0.284	1.125	0	7.18	1067
Total bile acids	2.081	2.932	0	24.7	1067
Total bilirubin	7.173	6.48	0	29.3	1067
Total protein	43.034	34.602	0	81	1067
Total oxygen saturation	0.043	0.807	0	16.5	1067
Total hemoglobin	0.013	0.43	0	14.038	1067
Arterial-to-alveolar oxygen partial pressure ratio at patient’s body temperature	0.234	4.457	0	98.6	1067
Alveolar-arterial oxygen pressure difference at patient’s body temperature	0.04	0.907	0	23.5	1067
Partial pressure of oxygen at patient’s body temperature	0.2	3.791	0	82.6	1067
pH at patient’s body temperature	0.021	0.392	0	7.427	1067
Anti-O	7.163	50.417	0	730	1067
Antithyroid peroxidase antibody	0.503	5.866	0	164.9	1067
Novel coronavirus nucleic acid test	0.003	0.037	0	0.5	1067
Immature reticulocyte index	0.004	0.129	0	4.2	1067
High-fluorescence reticulocytes	0.001	0.015	0	0.4	1067
Standard alkaline residue	-0.018	0.364	-9.5	0	1067
Standard bicarbonate	0.055	1.039	0	20.8	1067
Specific gravity	0.024	0.154	0	1.028	1067
Hydrogen ion	0.113	2.129	0	43.9	1067
Partial pressure of oxygen	0.256	4.214	0	82.6	1067
Oxyhemoglobin content	0.261	4.922	0	94.2	1067
Oxygen saturation	0.352	5.749	0	95.3	1067
Partial pressure of oxygen at 50% oxygen saturation	0.074	1.392	0	28.57	1067
Chloride	60.561	51.877	0	112	1067
Chloride ion concentration	0.449	7.346	0	131	1067
Activated partial thromboplastin time (APTT)	0.252	3.125	0	41.1	1067
Activated partial thromboplastin time control	0.259	2.985	0	35.9	1067
Percentage of lymphocytes	28.239	23.989	0	81.8	1067
Lymphocyte count	1.855	1.622	0	6.28	1067
Free triiodothyronine	1.154	2.591	0	9.54	1067
Free thyroxine	2.963	6.651	0	31.99	1067
Globulin	16.105	13.294	0	39.4	1067
Triglycerides	0.054	0.252	0	4.57	1067
Parathyroid hormone	0.073	0.568	0	6.9	1067
Thyroglobulin	0.282	2.359	0	46.7	1067
Thyroglobulin antibody	0.879	13.419	0	405	1067
Thyroxine	17.824	40.35	0	173.3	1067
WBC/PLT ratio	1.05	0.878	0	3.02	1067
White blood cell count	4.178	3.643	0	19.18	1067
Albumin	26.929	21.64	0	54.8	1067
Cortisol	6.196	45.943	0	513.2	1067
Direct bilirubin	2.187	1.963	0	9.3	1067
Alkaline phosphatase	148.803	142.242	0	687	1067
Carboxyhemoglobin	0.003	0.053	0	1.1	1067
Phosphorus	0.948	0.819	0	2.16	1067
Rheumatoid factor	0.048	0.447	0	7.2	1067
Red blood cell distribution width-CV	7.855	6.238	0	17.5	1067
Red blood cell distribution width-SD	23.564	18.714	0	46.5	1067
Hematocrit	24.865	19.763	0	50.1	1067
Erythrocyte sedimentation rate (ESR)	0.051	0.659	0	14	1067
Red blood cell count	3	2.391	0	6.28	1067
Fibrinogen (Stago)	0.014	0.179	0	2.79	1067
Vitamin B12	3.761	50.875	0	799.5	1067
Vitamin D	1.121	5.226	0	32.8	1067
Percent reticulocytes	0.003	0.063	0	1.57	1067
Absolute reticulocyte count	0	0.003	0	0.0799	1067
Creatinine	22.039	18.862	0	61	1067
Creatine kinase	74.903	105.619	0	2482	1067
Creatine kinase isoenzymes	11.957	11.194	0	69	1067
Alveolar-arterial oxygen pressure difference	0.04	0.907	0	23.5	1067
Alveolar oxygen partial pressure	0.24	4.517	0	86.8	1067
Glucose	1.686	2.343	0	7.6	1067
Glucose POCT	0.014	0.275	0	6.4	1067
Glucose concentration	0.017	0.286	0	5.214	1067
Platelet distribution width	6.566	5.464	0	19.2	1067
Platelet volume index	0.166	0.14	0	0.52	1067
Platelet count	175.302	148.586	0	521	1067
Oxygen-carrying capacity	0.045	0.847	0	17.6	1067
Blood ammonia	3.447	7.784	0	84.3	1067
Total carbon dioxide in plasma	0.115	2.182	0	44.1	1067
Hemoglobin	84.964	67.577	0	168	1067
Complement C3	0.014	0.118	0	1.35	1067
Complement C4	0.003	0.026	0	0.48	1067
Apolipoprotein A	0.092	0.36	0	1.77	1067
Apolipoprotein B	0.044	0.176	0	1.27	1067
Hyaluronic acid	0.009	0.066	0	0.5	1067
pH	0.028	0.453	0	7.4476	1067
Calcium	1.446	1.226	0	2.79	1067
Calcium ion concentration	0.004	0.094	0	2.75	1067
Sodium	80.865	69.241	0	145	1067
Potassium	2.505	2.158	0	5.7	1067
Potassium ion concentration	0.003	0.111	0	3.6328	1067
Iron (Whole Blood)	39.598	124.938	0	523.6	1067
Lead	3.242	10.685	0	80.98	1067
Copper (Whole Blood)	0.093	0.296	0	1.35	1067
Ceruloplasmin	0.04	0.101	0	0.43	1067
Zinc (Whole Blood)	0.426	1.365	0	7.23	1067
Magnesium	0.505	0.44	0	1.81	1067
Indirect bilirubin	4.986	4.664	0	23.3	1067
Fungi	0	0.015	0	0.5	1067
Fungi	0.012	0.076	0	0.5	1067
Yeast-like cells count	0.009	0.066	0	0.5	1067
Non-squamous epithelial cells	0.022	0.271	0	5	1067
Squamous epithelial cells	0.014	0.144	0	3	1067
Mucus	0	0.015	0	0.5	1067
Crystals	0.009	0.066	0	0.5	1067
Calcium oxalate crystals	0.009	0.066	0	0.5	1067
Pus cells	0	0.015	0	0.5	1067
Mucus filament quantity	0.01	0.076	0	1	1067
Pathological casts	0.009	0.066	0	0.5	1067
Trichuris eggs	0	0.015	0	0.5	1067
Hookworm eggs	0	0.015	0	0.5	1067
Ascaris eggs	0	0.015	0	0.5	1067
Pinworm eggs	0	0.015	0	0.5	1067
Trichomonas	0.012	0.076	0	0.5	1067
Tapeworm eggs	0	0.015	0	0.5	1067

Continuous variables are summarized as mean (SD) and range (minimum–maximum). Binary variables were coded as 0/1; therefore, the mean of a binary variable represents its prevalence (proportion with the feature present).

AUPRC = Area Under the Precision–Recall Curve, AUROC = Area Under the Receiver Operating Characteristic Curve, BECT = benign epilepsy with centrotemporal spikes, DCA = decision curve analysis, EEG = electroencephalogram, F1-score = F1 score (harmonic mean of precision and recall), KNN = k-nearest neighbors, LASSO = Least Absolute Shrinkage and Selection Operator, LDH = Lactate Dehydrogenase, MICE = Multivariate Imputation by Chained Equations, ML = machine learning, MLP = Multi-Layer Perceptron, PR = precision–recall, ROC = receiver operating characteristic, SeLECTS = self-limited epilepsy with centrotemporal spikes, SHAP = SHapley Additive exPlanations, SVM = support vector machine, VITD-T = total 25-hydroxyvitamin D, XGBoost = Extreme Gradient Boosting.

### 2.7. Final model and interpretative analysis

#### 2.7.1. Consistent with results

In a cross-comparison of ten models, XGBoost demonstrated optimal performance (internal validation AUROC ≈ 0.97, etc), thereby being selected as the final application prototype. Feature importance and directional explanations for XGBoost were obtained using SHapley Additive exPlanations (SHAP), with interpretative plots provided at both global and (exemplary) individual levels. Key features consistent with the results (e.g., temporal region, age, central, LDH, spikes) were highlighted to demonstrate their contribution order and directional influence in model decision-making. Decision curve analysis and calibration curves were presented for both internal and external datasets, mirroring the “test set/validation set” presentation style in the results section.

## 3. Results

### 3.1. Patient characteristics

**Figure 1.**
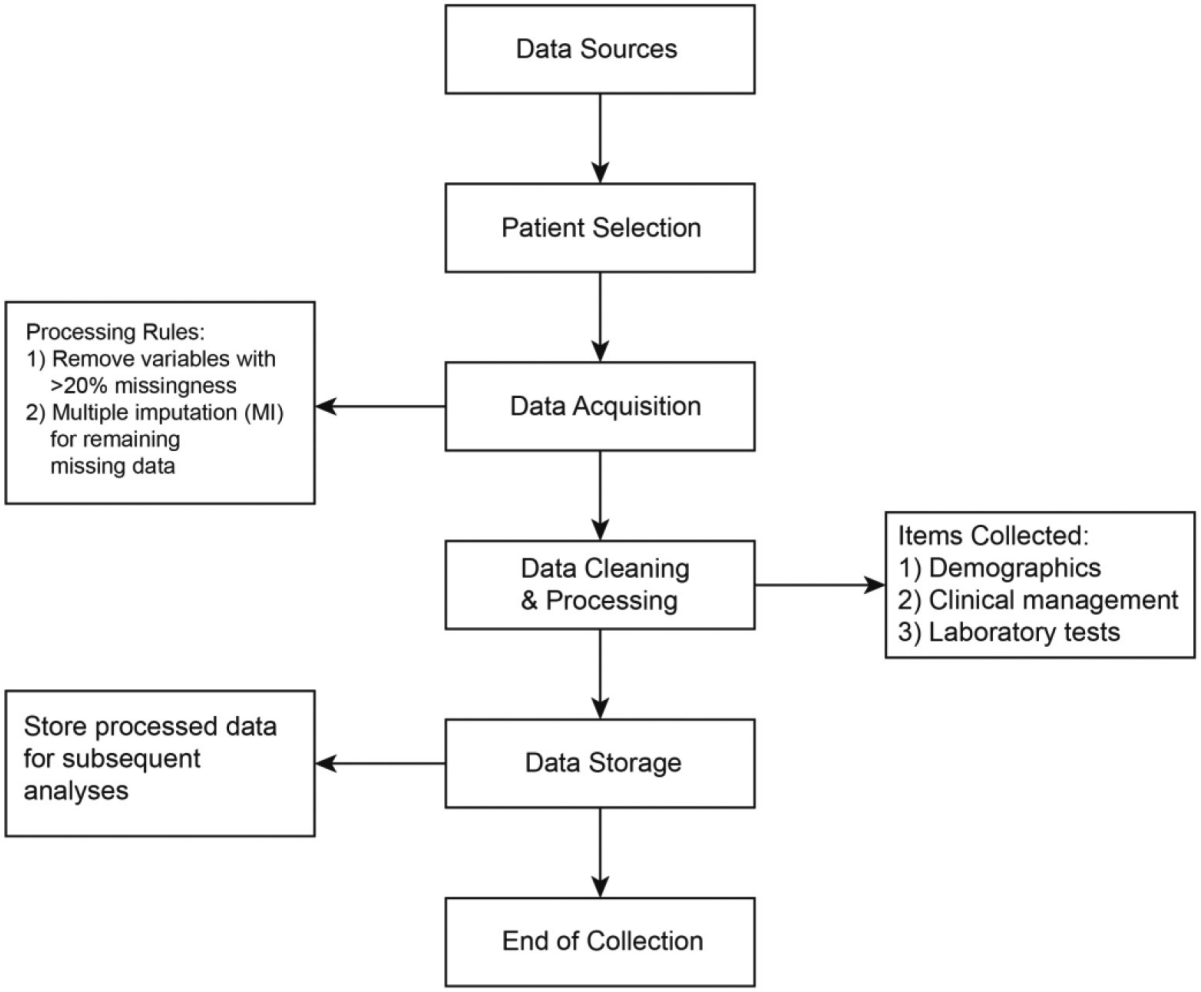
Data collection flowchart.

Based on the inclusion criteria (Fig. 1), a total of 1067 pediatric EEG-evaluated cases from our hospital were included for model development and internal evaluation, with SeLECTS/BECT as the target outcome and non-SeLECTS cases serving as the comparator class (Table 1). Additionally, 94 cases from other hospitals were included for external validation to assess cross-institutional generalizability. Table 1 provides numeric descriptive values for the study population (mean, standard deviation, minimum/maximum, and sample size for each variable). For binary EEG-report features encoded as 0/1, the reported mean equals the prevalence (e.g., male sex 0.59; central region involvement 0.79; temporal region involvement 0.823; spike 0.966). Age was extracted in days (mean 2847.123; range 317 to 6041).

### 3.2. Feature selection

**Figure 2.**
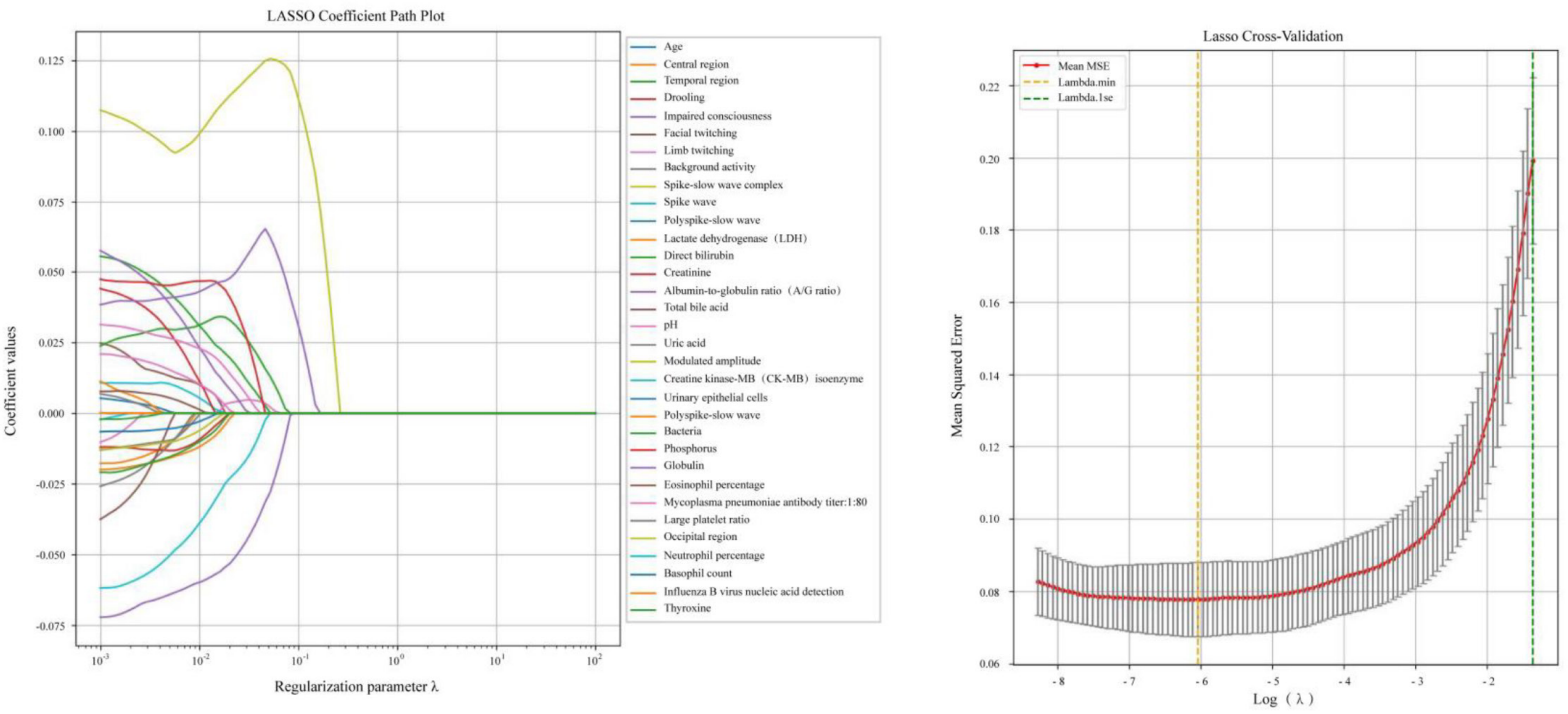
LASSO regression coefficient path map and LASSO regression cross-validation curve identified the most relevant feature columns through BECT clinical basic information and LASSO regression: Age, Central, Temporal Region, Facial Seizures, Limb Seizures, Background, Slow-Spike Complex, Spikes, Lactate Dehydrogenase, Eosinophil Percentage, Neutrophil Percentage, Basophil Count, 25-Hydroxyvitamin D (VITD-T).

We screened 242 features to identify the 13 most relevant ones using LASSO regression for feature selection. These features not only reflect patients’ clinical manifestations but also incorporate biomarkers and other relevant indicators. The coefficient path map and cross-validation curve from LASSO regression reveal the clinical background information associated with BECT, along with the most relevant feature columns selected by LASSO regression: Age, Central, Temporal, Facial Seizures, Limb Seizures, Background, Spike-and-Slow-Wave Complexes, Spikes, Lactate Dehydrogenase, Eosinophil Percentage, Neutrophil Percentage, Basophil Count, and 25-Hydroxyvitamin D (total 25-hydroxyvitamin D). These features provide a crucial foundation for subsequent modeling, effectively aiding in predicting BECT occurrence (Fig. 2).

### 3.3. Model performance comparison

In this study, we employed 9 ML models to diagnose patients with self-limiting central-temporal epileptiform spikes (SeLECTS, traditionally termed BECT). The predictive efficacy of each model was evaluated using receiver operating characteristic (ROC) curves and PR curves. Specifically, Logistic regression and Support Vector Machine both achieved an AUROC of 0.97 with accuracy rates of 0.93 and 0.94, respectively, demonstrating excellent performance. The decision tree model performed relatively weakly, with an AUROC of 0.83 and accuracy of 0.85. In contrast, the random forest model demonstrated improved diagnostic capability, achieving an AUROC of 0.96 and accuracy of 0.89.

In comparison, the XGBoost model demonstrated the best overall predictive performance. This model excelled across multiple metrics: an AUROC of 0.97, accuracy of 0.91, sensitivity of 0.83, specificity of 0.94, and an F1 score of 0.83, revealing its robust capability in diagnosing BECT patients. Additionally, the Multi-Layer Perceptron model achieved an AUROC of 0.98, accuracy of 0.93, and sensitivity of 0.88, indicating favorable performance. Other models such as Gradient Boosting (AUROC 0.98, accuracy 0.92) and k-Nearest Neighbors (AUROC 0.96, accuracy 0.92) also demonstrated robust diagnostic capabilities, providing effective support for the clinical diagnosis of BECT.

### 3.4. XGBoost model performance validation

**Figure 3.**
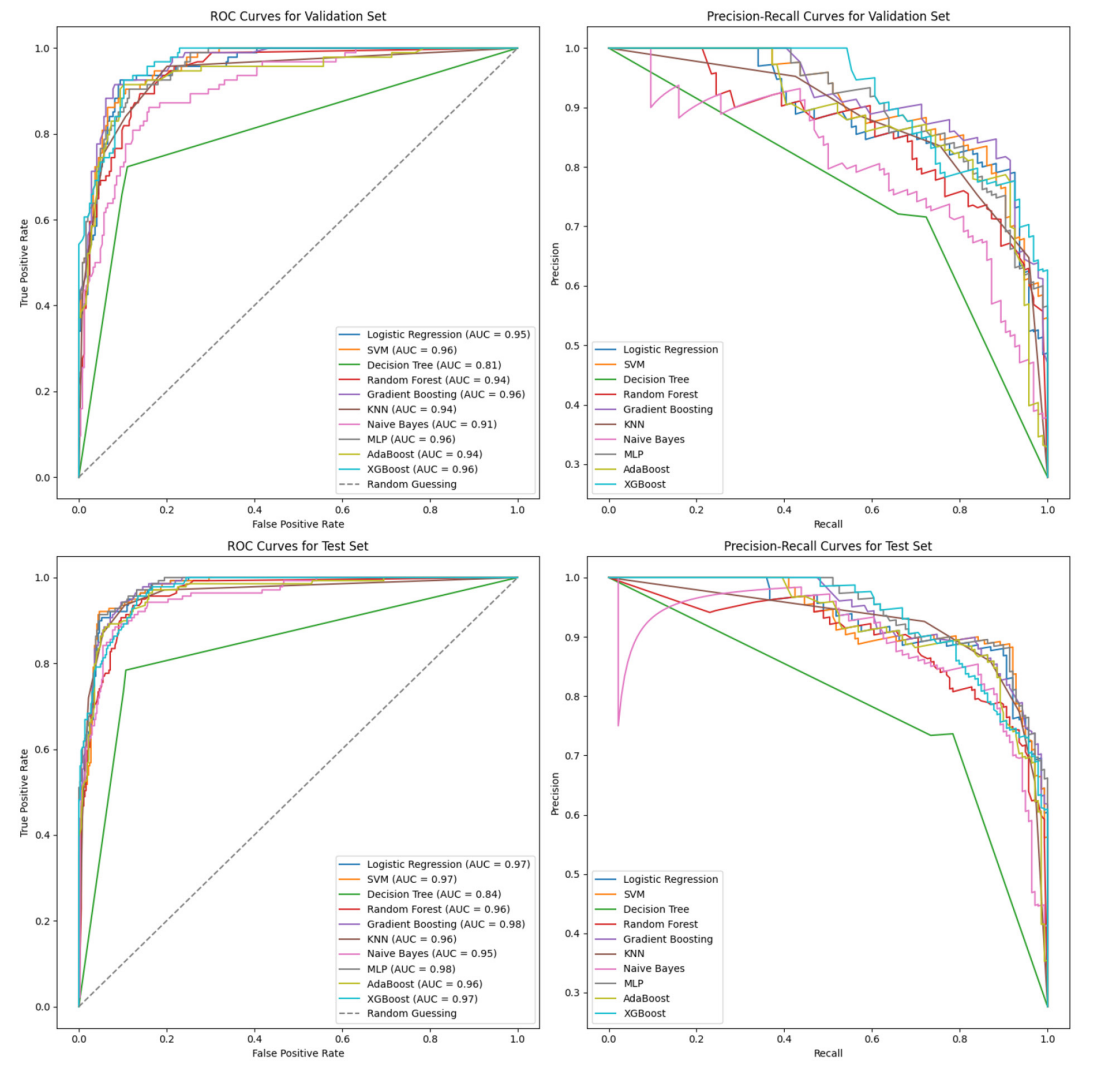
Display the performance of multiple classification algorithms on the validation and test sets using ROC curves (left column) and PR curves (right column). These algorithms include Logistic Regression, Support Vector Machine (SVM), Decision Tree, Random Forest, Gradient Boosting, k-Nearest Neighbors (kNN), Naive Bayes, Multi-Layer Perceptron (MLP), AdaBoost, and XGBoost. Gradient Boosting and XGBoost models performed exceptionally well on both the validation and test sets, exhibiting high AUC values and an excellent precision-recall balance, making them the preferred model choices.

**Figure 4.**
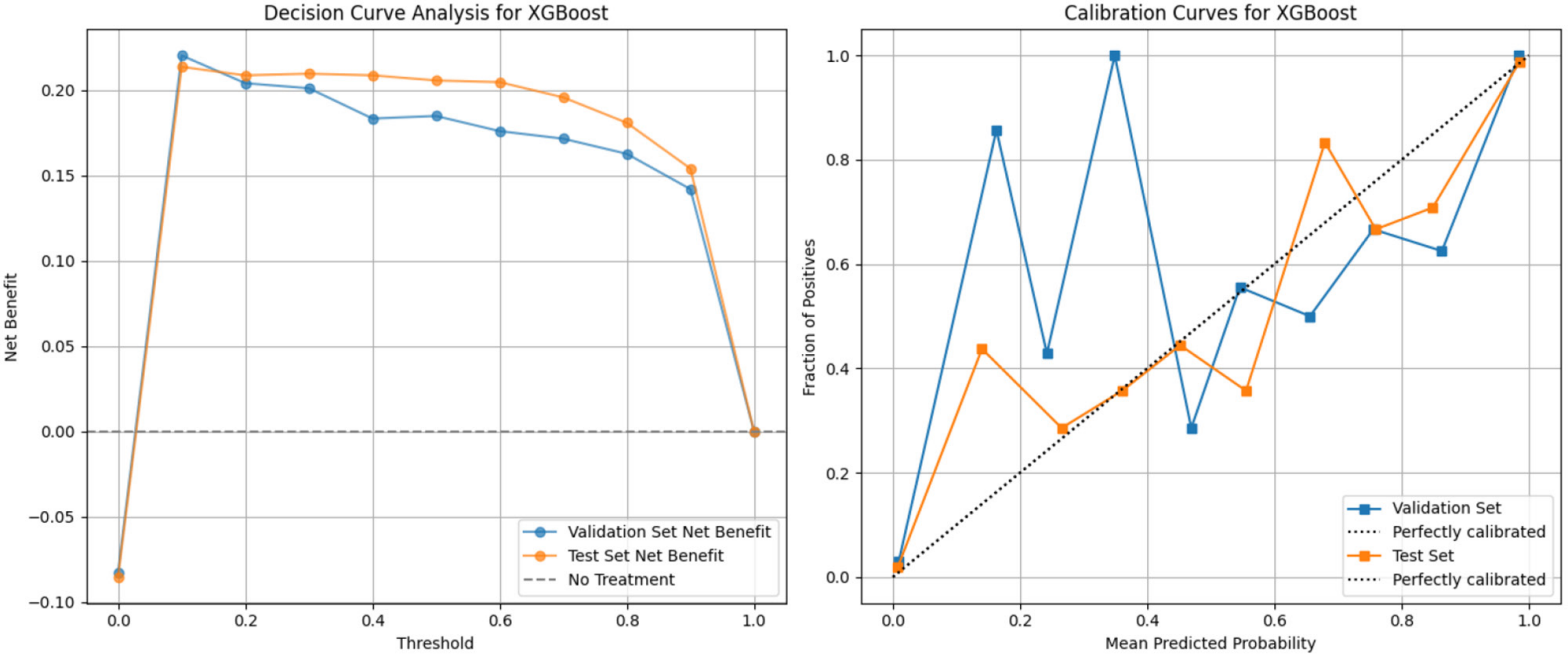
The net benefit is positive at most thresholds in the test/validation set, and the predicted probability generally aligns with the actual positive rate.

Combined results from the ROC curve and PR curve demonstrate that the XGBoost algorithm exhibits high recognition accuracy in this study, enabling more precise classification of BECT patients. We selected this algorithm for application development (Figs. 3 and 4).

### 3.5. External validation of XGBoost model performance

**Figure 5.**
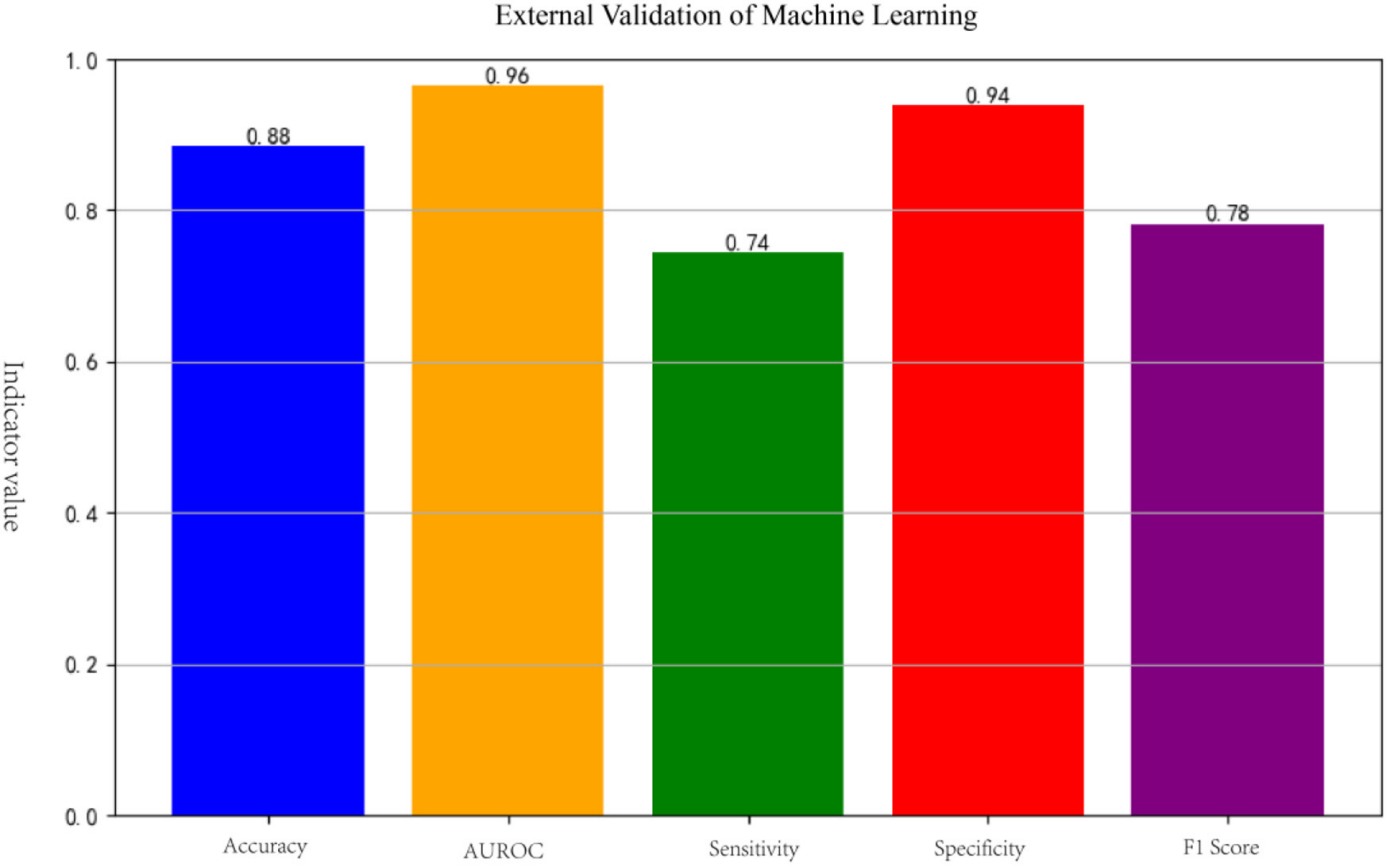
External validation results of the XGBoost Model.

In this study, we conducted external data validation on the trained XGBoost model to assess its generalization performance on new patient data from other hospitals. Validation results indicate the model achieved an overall prediction accuracy of 88% across the sample, demonstrating robust classification performance and high practical value. The model’s Area Under the ROC Curve (AUROC) reached 96%, reflecting its excellent discrimination capability between positive and negative samples. The model’s sensitivity was 74%, indicating strong detection capability for the target class. Specificity reached 94%, demonstrating outstanding recognition of negative samples. With an F1 score of 78%, the model achieved a favorable balance between precision and recall for positive samples, further validating its reliable classification performance (Fig. 5).

External validation results indicate that the XGBoost model exhibits robust performance on new data, effectively supporting sample classification and identification.

### 3.6. Application of SHAP plots

**Figure 6.**
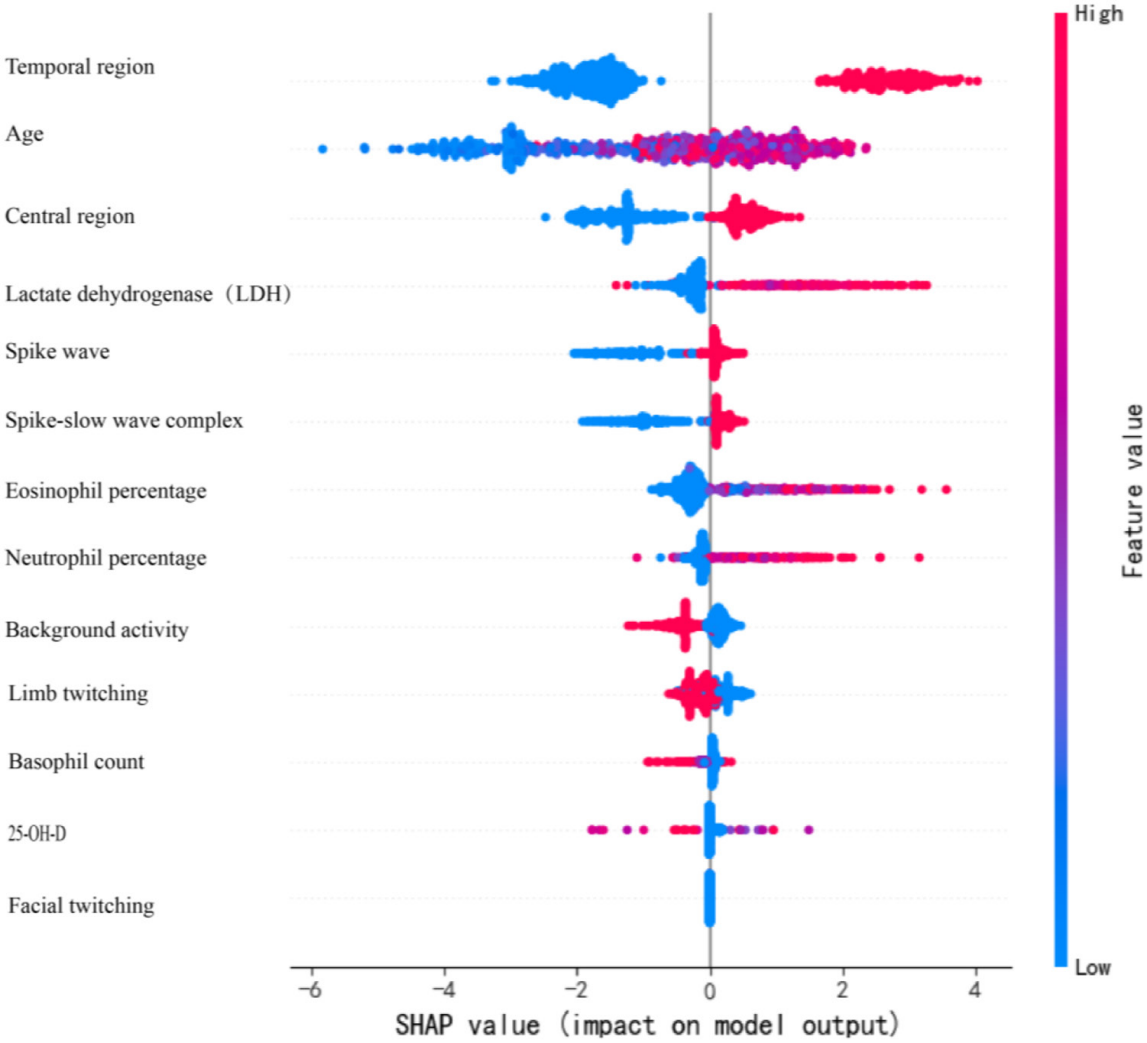
The role and importance of features in the model’s prediction of BECT. Temporal region, age, central, lactate dehydrogenase, and spike are key features for the model’s BECT prediction. High values typically support the BECT prediction. Other indicators have some influence, but their contribution to the prediction outcome is relatively minor compared to the key indicators.

This study employs the SHAP method to analyze feature importance in ML models, aiming to gain deeper insights into each feature’s contribution to BECT prediction outcomes. The SHAP plot (Fig. 6) illustrates the role and significance of various features when the model predicts BECT. SHAP values provide an intuitive way to identify which features most significantly impact the model’s predictions. The temporal region, age, central, lactate dehydrogenase, and spike are key features for BECT prediction. High values of these features typically correlate positively with BECT predictions, indicating their crucial role in the model’s decision-making. While other indicators also exert some influence, their contribution to prediction outcomes is relatively minor compared to these key features.

The SHAP plots further validate the features selected via LASSO regression in this study, demonstrating the interpretability of ML models. This aids in understanding how to make more precise BECT predictions based on clinical data in practical applications.

### 3.7. Example of web tool usage

**Figure 7.**
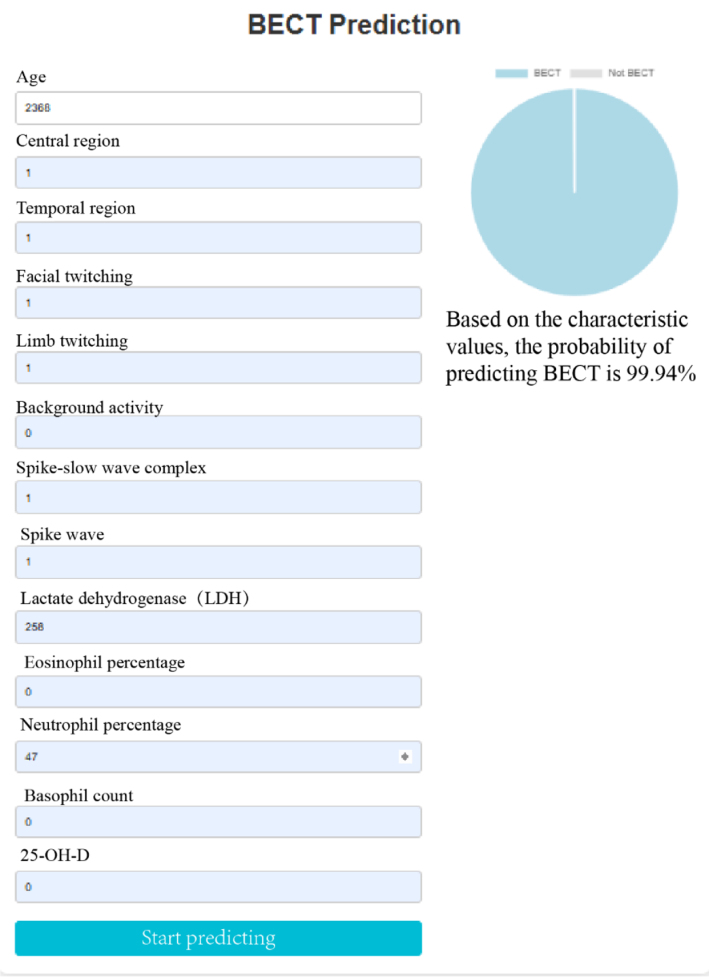
Example of using web tools.

Figure 7 demonstrates an example of using the web tool based on the ML model developed in this study. The tool provides clinicians with convenient support for predicting BECT. By inputting 13 structured features of a suspected SeLECTS patient, users can quickly obtain the probability of that patient being diagnosed with BECT.

In the example, clinical data from a BECT patient is entered, and the system instantly calculates and displays the probability of an EEG diagnosis confirming BECT. The results indicate an extremely high probability of BECT diagnosis for this patient. This functionality not only enhances clinical decision-making efficiency but also provides scientific evidence for physician patient management.

The web tool’s design prioritizes user experience with an intuitive interface and straightforward operation, enabling clinicians to rapidly apply ML models in practice and thereby enhance BECT identification and management capabilities. Future research may further optimize this tool by adding more clinical parameter input options and expanding its applicability to better serve clinical needs.

## 4. Discussion

This study utilized real-world population data from multiple centers (internal *n* = 1067, external *n* = 94) to construct a ML model for the auxiliary identification of SeLECTS (BECT) syndrome based on structured EEG reports and routine laboratory information dimensions.^[22]^ LASSO screening identified 13 features with high information density and cross-center reusability from 242 candidate variables, followed by systematic comparison across ten common algorithms.^[23,24]^ In the head-to-head comparison, XGBoost demonstrated the most robust overall profile and retained stable performance on independent external validation, supporting its selection as the final application prototype. Decision curve and calibration analyses further suggested clinically meaningful net benefit and good agreement between predicted and observed risks across commonly used threshold ranges in practice.^[25]^The calibration curve indicates that the model’s predicted probabilities align with actual observed risks, forming a continuous evidence chain of “discrimination-calibration-net benefit” that meets the fundamental requirements of “discerning, accurately distinguishing, and distinguishing with value.”

This high-level model performance demonstrates the significant application potential of ML in EEG-based syndrome identification, consistent with previous multicenter studies showing high accuracy in automated EEG analysis and effective clinical decision support.^[26–28]^ In prior work, gradient-boosted tree approaches and other ML pipelines were primarily evaluated in broader seizure detection/prediction tasks or general automated EEG interpretation across heterogeneous neurological contexts; these settings differ from syndrome-level classification and thus offer a useful but imperfect comparator for SeLECTS.^[26–28]^ Notably, SeLECTS, a common pediatric epilepsy syndrome, is frequently underdiagnosed or misdiagnosed clinically due to predominantly nocturnal seizures and transient, cryptic symptoms.^[29,30]^ By focusing on a syndrome with distinct age dependence and characteristic centrotemporal spike morphology, our findings suggest that XGBoost can leverage structured report-level descriptors to capture syndrome-specific signatures without requiring raw waveform processing, thereby enabling real-time quantitative risk assessment to support clinical decision-making.

### 4.1. Biological interpretation and feature selection

From pathophysiological and clinical electrophysiological perspectives, top-ranked features in the model’s interpretive results include central-temporal region entries and morphological elements such as spikes and spike-wave complexes.^[31,32]^ These findings align closely with the classic EEG topography and morphological characteristics of SeLECTS.^[33,34]^ The most characteristic EEG manifestation of SeLECTS (benign childhood central-temporal epilepsy) is high-amplitude sharp spikes in the central region, often accompanied by subsequent slow waves, which are more pronounced during sleep.^[35,36]^

This finding indicates that even when relying solely on standardized reporting-level variables: without direct access to raw EEG waveforms: the model can capture the core electrophysiological features of this syndrome across multicenter data. The high weight assigned to “age” in the model aligns with clinical intuition: SeLECTS predominantly manifests in school-aged children (approximately 3 to 14 years old, with a peak incidence between 7 to 10 years).^[37,38]^ Age also intertwines with observable factors such as sleep-related discharge burden and background rhythms, making the “age × EEG phenotype” a key axis in diagnostic reasoning.

It is crucial to emphasize that certain laboratory metrics included in the model (e.g., LDH, white blood cell differential count) are not direct epileptogenic biomarkers. Their contribution likely reflects indirect influences on “identification” as SeLECTS: such as the patient’s clinical state at presentation and timing of testing: and should thus be regarded as proxy signals reflecting clinical trajectories rather than direct therapeutic targets or etiologic evidence.^[37]^

### 4.2. Algorithm selection and external validation

XGBoost was selected as the final model not only for its outstanding discriminative capability but also for its consistent performance in external validation.^[38]^ The model exhibited only a slight decline in performance on external data (slightly reduced sensitivity, largely unchanged specificity), with decision curves indicating it still provided a net benefit similar to that observed internally.^[25,38]^ Consequently, under the “model-based initial screening + manual review” workflow, the model continues to offer substantial decision-making value for identifying high-risk patients.

Furthermore, the model utilizes only 13 structured features, offering good interpretability while avoiding complex signal processing and high computational demands, facilitating lightweight deployment across multiple centers.^[39–41]^

### 4.3. Threshold setting and workflow integration

Model thresholds should balance the risk of missed diagnoses against the burden of follow-up examinations based on specific clinical scenarios. Lower thresholds reduce missed diagnoses (increasing sensitivity) but increase follow-up workload, while higher thresholds have the opposite effect. Given that decision curves indicate stable net benefits even at low-to-moderate thresholds,^[25]^ integrating threshold settings with quality control processes is recommended. Within EEG reporting interfaces, concise risk cards can display model-generated risk scores alongside key feature alerts. When model judgments diverge from initial manual readings, automatic triggers for secondary reviews or team discussions can be implemented.^[41]^

Through this “human-machine co-review” model, the system can evolve from a simple “scorer” to a “controller” that assists quality control: enhancing reading consistency while providing feedback examples and maximizing clinical workflow value.^[41]^

### 4.4. Model interpretation and clinical acceptance

Regarding model interpretation, it is crucial to note that feature importance does not equate to causality. Features with high importance scores in SHAP merely indicate factors relevant to the model’s decision-making process, not direct causes of disease.^[42]^ Therefore, top-ranked features in the model should be treated as supportive interpretive evidence rather than proof of disease mechanisms. When communicating with clinicians, clear, concise, and clinically relevant explanations should be provided to build trust while avoiding overly complex interpretations, balancing confidence and skepticism.^[43]^

## 5. Limitations and Future Directions

While the findings of this study are encouraging, several limitations warrant attention. The model data primarily originate from a single region, and the external validation sample size remains limited; its performance across broader populations and diverse subgroups has yet to be verified.^[38,39]^ Additional data from multiple centers are needed to assess generalization and stability. The current model relies on static retrospective data, failing to capture dynamic changes during disease progression; future studies should incorporate follow-up or time-series analyses to enhance adaptability.^[39,40]^

In summary, we established and externally validated an interpretable and deployable SeLECTS auxiliary identification model based on 13 structured, cross-center reusable features. The model is intended as a post-reporting “second view”: it outputs a patient-level probability and transparent feature cues that can prompt targeted rereview when the model output diverges from the initial EEG impression, thereby supporting triage and follow-up stratification across centers. With ongoing prospective validation and recalibration, this approach is feasible for multicenter deployment and may facilitate standardized, quality-controlled syndrome recognition in pediatric epilepsy practice.^[41,43]^

## 6. Conclusion

We constructed an auxiliary recognition model for SeLECTS using a streamlined and interpretable feature set across multicenter populations, validating its comprehensive value in discrimination, calibration, and net decision benefit on external independent data. The model is not intended to replace diagnosis; instead, after routine EEG reporting it provides a quantitative SeLECTS probability and an interpretable summary of the key report-level drivers. This enables neurologists to prioritize second reads or additional confirmation when clinically indicated in flagged cases, and to standardize triage and tiered review across centers. In resource-constrained and labor-limited settings, this “risk alert + interpretable cues” workflow may help mitigate missed or delayed syndrome recognition while preserving clinician oversight, providing a practical foundation for prospective validation and cross-platform deployment.

## Author contributions

**Data curation:** Lijun Li, Lei Li.

**Conceptualization:** Lingxiang Ao.

**Formal analysis:** Lei Li.

**Writing – original draft:** Lijun Li, Li Wang.

**Writing – review & editing:** Xiaomei Liu, Kai Liu.
